# Inhibition of CYP450 family 1 subfamily B member 1 (CYP1B1) expression in macrophage reduces the inflammatory response in type 2 diabetes mellitus combined with tuberculosis

**DOI:** 10.3389/fendo.2025.1617292

**Published:** 2025-08-21

**Authors:** Qianqian Du, Kun Liu, Yanling Li, Xinyan Wang, Xin Liu, Jing Zhao, Xuemei Wang

**Affiliations:** School of Public Health, Inner Mongolia Medical University, Huhhot, China

**Keywords:** tuberculosis, type 2 diabetes, macrophages, CYP1B1, inflammatory response

## Abstract

Type 2 diabetes (T2DM) and tuberculosis (TB) both regulate inflammation and may exert synergistic or antagonistic effects through shared immune pathways. Previous studies have demonstrated that T2DM is a risk factor for TB. However, at the level of gene regulatory networks, it remains unclear whether there are key interaction nodes linking these two diseases. In this study, we integrated bioinformatic analysis from the Gene Expression Omnibus (GEO) database and performed differential gene expression analysis and weighted gene co-expression network analysis (WGCNA). Furthermore, we applied machine learning techniques to identify key genes among the commonly differentially expressed genes (DEGs). In addition, this study employed siRNA in THP-1 cells to validate the cross-talk genes selected through bioinformatic analysis. The THP-1 cells were treated with high-concentration glucose (15.5 μM, Glu), *Mycobacterium tuberculosis* ESAT-6, or Glu+ESAT-6. We identified a total of 23 common genes between TB and T2DM using DEGs and WGCNA. Furthermore, expression patterns from external datasets revealed three key cross-talk genes linking TB-T2DM: *CYP1B1*, *SERPING1*, and *CHPT1*. Notably, only *CYP1B1* was significantly upregulated in the THP-1 detection test, compared to the unstimulated (control) group (*P* < 0.05). Moreover, *CYP1B1* significantly reduced the expression of pro-inflammatory cytokines (TNF-α, IL-6, IL-1β, IL-10), M2 macrophage polarization markers (CD163, Arg-1), and chemokines (CXCL-10), and was associated with the NOD2 and TRAF6 signaling pathways (*P* < 0.05). These findings elucidate the regulatory mechanisms underlying the comorbidity of TB and T2DM, providing a theoretical basis for the development of precise combination therapies and novel therapeutic targets.

## 1 Introduction

Tuberculosis (TB) and Type 2 Diabetes Mellitus (T2DM) are chronic diseases with significant public health implications worldwide. The comorbidity of TB and T2DM (TB-T2DM) represents a growing concern for public health, particularly in countries with high disease prevalence, such as India, China, and Indonesia (1). According to the latest data from the World Health Organization (WHO), approximately 10.8 million new cases of TB were reported worldwide in 2023, marking an increase of 0.93% compared to 2022 (2). Simultaneously, the prevalence of T2DM is rising sharply, largely due to unhealthy dietary habits and increasing rates of obesity, particularly in developing countries (3). The co-occurrence of these diseases not only severely impairs patients’ quality of life but also imposes a considerable burden on healthcare systems (4).

Epidemiologically, the interaction between T2DM and TB results in a more severe clinical course for diabetic patients (5). Individuals diagnosed with T2DM are at a threefold increased risk of developing TB compared to those without diabetes. In high-burden regions, approximately 21% of TB patients are comorbid with T2DM (6). Studies have demonstrated that patients suffering from both TB and T2DM experience higher rates of treatment failure (RR=1.69, 95% CI 1.36–2.12), disease recurrence (RR=3.89, 95% CI 2.43–6.23), and all-cause mortality (RR=1.89, 95% CI 1.52–2.36) when compared to individuals with TB alone (6–8). These findings underscore the complex interplay between TB and T2DM and the significant impact of this comorbidity on clinical outcomes.

Although significant progress has been made in understanding the independent pathogenesis of TB and T2DM, research on their interactions, particularly at the immunological and inflammatory levels, remains limited. For instance, T2DM disrupts immune function, resulting in dysregulated inflammatory responses. Recent studies have shown that oxidative stress in patients with T1DM leads to significant DNA damage and a concurrent reduction in the efficacy of the antioxidant defense system (9). Furthermore, it has been observed that individuals with diabetes exhibit increased levels of pro-inflammatory cytokines, such as interleukin-6 (IL-6) and tumor necrosis factor-α (TNF-α), which contribute to chronic inflammation, exacerbating pancreatic β-cell damage and insulin resistance (10, 11). T2DM impairs the immune response to TB, reducing the ability to clear *Mycobacterium tuberculosis*. These complications may be exacerbated by altered inflammatory responses, as elevated levels of inflammatory cytokines in T2DM can influence TB pathogenesis and hinder effective treatment outcomes (12). Macrophages, which play a crucial role in initiating immune responses by phagocytosing TB bacteria and secreting key cytokines such as TNF-α and IL-6, demonstrate impaired functionality in individuals with diabetes due to the presence of chronic, low-grade inflammation (13, 14). Restrepo et al. demonstrated that patients with TB-T2DM exhibit more intense immune responses compared to patients with TB alone, with elevated levels of interferon-γ (IFN-γ), interleukin-2 (IL-2), TNF-α, and granulocyte-macrophage colony-stimulating factor (GM-CSF) (15). Conversely, TB, caused by *M. tuberculosis*, elicits a strong immune response marked by the activation of immune cells and increased production of pro-inflammatory cytokines such as TNF-α, IL-1β, and IL-6. These cytokines are pivotal in immune evasion and the chronicity of TB (16). Excessive inflammatory responses can exacerbate TB lesions and compromise overall immune function (17). Conversely, persistent TB-driven inflammation and immune activation may exacerbate metabolic dysregulation in T2DM, thereby worsening insulin resistance and glycemic control. This bidirectional mechanism creates a vicious cycle: T2DM-induced immunosuppression provides an ideal environment for *M. tuberculosis* survival, while TB-related chronic inflammation exacerbates diabetic metabolic disturbances (18–23). Systems biology analyses have revealed significant dysregulation of key immune pathways, including the Jak-STAT and NF-κB pathways, in patients with comorbid diabetes and tuberculosis (24). However, the interaction mechanisms between these two diseases at the level of gene regulatory networks remain unclear.

In summary, both T2DM and TB induce systemic inflammatory responses, exhibiting either synergistic or antagonistic interactions in their inflammatory pathways. However, whether cross-talk genes within their gene expression networks that may regulate TB-T2DM-associated inflammatory and metabolic dysfunctions remains unexplored.

To address these questions, this study integrates bioinformatics analysis with experimental validation to identify core genes involved in inflammatory dysregulation and elucidate the molecular mechanisms underlying the TB-T2DM. By utilizing publicly available transcriptomic data from the GEO database, WGCNA, and machine learning algorithms, we identify common candidate genes associated with TB-T2DM. Functional enrichment and immune infiltration analyses are employed to validate the functions of these genes in inflammation and immune regulation, supported by qPCR and Western blot experiments.

The novelty of this study lies in its integrative approach, which combines bioinformatics analysis, machine learning techniques, and experimental validation to systematically identify cross-talk genes and pathways associated with inflammation in TB-T2DM. These findings offer new scientific insights into the inflammatory mechanisms underlying TB-T2DM comorbidity, laying the groundwork for the development of novel diagnostic biomarkers and targeted therapeutic strategies.

## 2 Materials and methods

### 2.1 Data collection and preparation

Transcriptomic datasets related to T2DM and TB were downloaded from the Gene Expression Omnibus (GEO) database (https://www.ncbi.nlm.nih.gov/geo/). The TB dataset (GSE28623), based on the GPL4133 platform, included blood samples from 46 TB patients and 37 control samples. The T2DM dataset (GSE166502), based on the GPL10558 platform, comprised myocyte samples from 13 T2DM patients and 13 controls. These datasets served as the training set. Additionally, GSE34608, comprising 8 TB blood samples and 18 control samples, was utilized as an independent validation cohort to evaluate the reliability and accuracy of the findings.

### 2.2 Identification of DEGs

Raw expression matrices were normalized, and DEGs between the TB and control groups in the GSE28623 dataset were identified using the limma package in R (25). Genes were selected based on an adjusted *P* < 0.05 and |logFC| ≥ 1. The “pheatmap” and “ggplot2” packages in R were used to generate clustering heatmaps and volcano plots of DEGs.

### 2.3 Weighted gene co-expression network analysis and identification of key modules

WGCNA is a bioinformatics method used to characterize gene co-expression patterns across different samples. This method clusters genes with similar expression profiles and explores the associations between identified modules and specific traits or phenotypes (26). To identify gene clusters associated with T2DM in the GSE166502 dataset, hierarchical clustering was performed using the “Hclust” function in R to detect outliers. Subsequently, the “pickSoftThreshold” function from the WGCNA package was used to calculate the soft threshold and adjacency matrix, with a criterion of R² > 0.8. The adjacency matrix was further converted into a topological overlap matrix (TOM) to measure gene associations by incorporating both direct and indirect interactions. The TOM matrix was converted into a dissimilarity matrix to generate a hierarchical clustering tree. Gene modules showing a statistically significant correlation with T2DM (*P* < 0.05, correlation coefficient > 0.6) were selected as key modules for further analysis.

### 2.4 Identification of shared genes and pathway enrichment

Venn diagrams were employed to identify overlapping genes between key T2DM modules and TB DEGs. Functional enrichment analysis of these shared genes was performed using the “ClusterProfiler” package in R for Gene Ontology (GO) and Kyoto Encyclopedia of Genes and Genomes (KEGG) pathway analysis (27). The results were visualized using the Sangerbox biomedical platform (28), with a *P* < 0.05 considered statistically significant.

### 2.5 Machine learning for key gene screening

The study comprehensively employed four machine learning algorithms—LASSO regression, random forest (RF), support vector machine (SVM), and Gaussian mixture model (GMM)—to screen for pivotal genes. LASSO Regression: The glmnet package was used with 10-fold cross-validation to determine the penalty parameter λ and identify significant genes (29). RF: The RF package was used to rank genes based on feature importance scores (30). SVM Recursive Feature Elimination (SVM-RFE): 5-fold cross-validation was used to identify the optimal feature gene set (31). GMM: Core key genes were identified through intersection analysis of the gene sets selected by the aforementioned methods (32).

### 2.6 Receiver operating characteristic curve and nomogram construction

The diagnostic performance of key genes was evaluated using RNA microarray data from the training set (46 TB patients and 44 controls). ROC curves were plotted for each gene, and the area under the curve (AUC) was calculated to evaluate diagnostic accuracy. The GSE34608 dataset was used for validation. Key genes exhibiting consistent expression profiles and an AUC greater than 0.7 in both the training and validation datasets were selected as characteristic biomarkers. Nomograms were constructed using the “RMS” package to predict TB occurrence by summing scores from cross-talk genes (33). The stability and predictive performance of the model were further evaluated through ROC analysis.

### 2.7 Single-sample gene set enrichment analysis

ssGSEA was performed using the “GSEA” R package to investigate functional and signaling pathways associated with diagnostic biomarkers. Significant pathways were selected based on normalized enrichment scores (NES), NOM p-value < 0.05, and FDR q-value < 0.05.

### 2.8 Immune infiltration analysis

The CIBERSORT algorithm was employed to estimate the proportions of immune cells from RNA expression data (34). GSE28623 datasets used whole-blood transcriptomes, meeting CIBERSORT’s requirement for total cellular RNA. A Spearman correlation analysis was conducted between the 22 immune cell types and cross-talk genes using the “corrplot” package. The correlation results were visualized through bar plots generated with the “ggplot2” package.

### 2.9 Cell culture and construction of an *in vitro* high-glucose TB infection model

The human monocytic leukemia cells (THP-1) were cultured in DMEM (Gibco, China) supplemented with 10% fetal bovine serum (FBS, Gibco, China) and 1% penicillin-streptomycin (Gibco, China) at 37°C under 5% CO_2_. The culture medium was replaced after 24 h. Based on previous studies by Baker and Alebel et al. (35, 36), THP-1 cells were stimulated with glucose at concentrations of 15.5 μM and 5.5 μM, ESAT-6 (an important pathogenic protein of *M. tuberculosis*, Gene Optimal, China), and 15.5 μM glucose combined with ESAT-6, as well as 5.5 μM glucose combined with ESAT-6, respectively.

### 2.10 siRNA transfection

THP-1 cells were placed in 6-well plates and differentiated into macrophages using PMA (MedChemExpress, USA). Cells were transfected with *CYP1B1*-siRNA or si-NC (MedChemExpress, USA) using Lipofectamine 2000 (Thermo Fisher, USA) in Opti-MEM medium (Gibco, China). The transfected cells were incubated for 24 hours before proceeding to further analysis.

### 2.11 qPCR for mRNA expression analysis

RNA extraction was carried out from cells after 8 hours of cell treatment, following the protocol provided by the Axygen RNA kit manufacturer (Axygen Scientific, USA). RNA (with an A260/A280 ratio between 1.8 and 2.1) was reverse transcribed into cDNA using the Primer Script™ RT Master Mix Kit (Takara, China). qPCR was performed using the ABI 7500 real-time PCR system (Bio-Rad, USA). The reaction conditions were as follows: an initial denaturation at 95°C for 10 minutes, followed by 40 cycles consisting of denaturation at 95°C for 15 seconds and annealing/extension at 60°C for 30 seconds (37). The 2^−ΔΔCt^ method was used to calculate relative gene expression levels. ΔCt1 was calculated as the difference between the β-actin CT value in the sample and the β-actin CT value in the control. Similarly, ΔCt2 was determined by subtracting the CT value of the target gene in the control from that in the sample. ΔΔCt was then calculated as ΔCt1 minus ΔCt2. (The RNA sequence fragments are presented in **Table 1**).

**Table 1:** PCR primers.

Gene	Forward primer sequence	Reverse primer sequence
*CHPT1*	*CCAGTTCTTGGATTTCTAGGTGG*	*CCTATGTGGAGTCCAGGTGACA*
*SERPING1*	*GCATCAAAGTGACGACCAGCCA*	*GTCTCTGTCAGTTCCAGCACTG*
*CYP1B1*	*GCCACTATCACTGACATCTTCGG*	*CACGACCTGATCCAATTCTGCC*
*IL6*	*ACTCACCTCTTCAGAACGAATTG*	*CCATCTTTGGAAGGTTCAGGTTG*
*IL10*	*GACTTTAAGGGTTACCTGGGTTG*	*TCACATGCGCCTTGATGTCTG*
*IL1β*	*ATGATGGCTTATTACAGTGGCAA*	*GTCGGAGATTCGTAGCTGGA*
*TNF-α*	*GAGGCCAAGCCCTGGTATG*	*CGGGCCGATTGATCTCAGC*
*CXCL-10*	*TTCCTGCAAGCCAATTTTGT*	*ATGGCCTTCGATTCTGGATT*
*β-ACTIN*	*CTGAACCCCAAGGCCAAC*	*AGCCTGGATAGCAACGTACA*
*CD163*	*ATTGCAGTCGGGATCCTTGG*	*CGCTGTCTCTGTCTTCGCTT*
*Arg-1*	*AACACTCCCCTGACAACCA*	*CATCACCTTGCCAATCCC*

### 2.12 Western blot for protein expression analysis

Proteins were collected after 12 hours for Western blot. Western blot was performed as previously described (38). Protein lysates (30 μg) were quantified using the Enhanced BCA Protein Assay Kit (Beyotime, China) and subsequently separated by SDS-PAGE (Solarbio, China) using a Mini-PROTEAN Tetra electrophoresis system (Bio-Rad, USA). The 30 μg protein lysates were standardized using the Enhanced BCA Protein Assay Kit (Beyotime, China) and separated by SDS-PAGE (Solarbio, China). Proteins were then transferred onto PVDF membranes (Immobilon, Germany) and probed with antibodies against *CYP1B1*, NOD2, TRAF-6, and β-actin (Proteintech, China). The *CYP1B1* and β-actin primary antibodies were diluted to 1:3000, NOD2 primary antibodies to 1:1000, and the TRAF-6 primary antibodies were diluted to 1:500 using the primary antibody dilution reagent (Beyotime, China). The samples were left to incubate overnight at 4°C. Afterward, the membranes underwent three washes with TBST (Solarbio, China), each lasting 10 minutes. Subsequently, the membranes were incubated with secondary antibodies (Cell Signaling Technology, USA) at a dilution of 1:3000 for 1 hour at room temperature, followed by four additional washes with TBST, each lasting 5 minutes. Finally, the immune-reactive proteins were visualized using an enhanced chemiluminescence reagent (ECL Western Blotting, Affinity Biosciences, USA) according to the manufacturer’s instructions.

### 2.13 ELISA for cytokine quantification

Cell culture supernatants were collected after 12 hours of cell treatment, and the concentrations of IL-6, IL-1β, and TNF-α were measured using ELISA kits (Lunchangshuo, China).

### 2.14 Statistical analysis

Bioinformatics analyses were performed using R version 4.3.3. Experimental data were analyzed using GraphPad Prism 6.1. Multiple group comparisons were conducted using one-way ANOVA with Tukey’s HSD test. For comparisons between two groups, either an independent samples t-test (for normally distributed data) or the Wilcoxon rank-sum test (for non-normally distributed data) was applied. A two-tailed *P* < 0.05 was considered statistically significant. The flow chart of this research is shown in **Supplementary Figure S1**.

## 3 Results

### 3.1 Identification of DEGs

**Figure 1:**
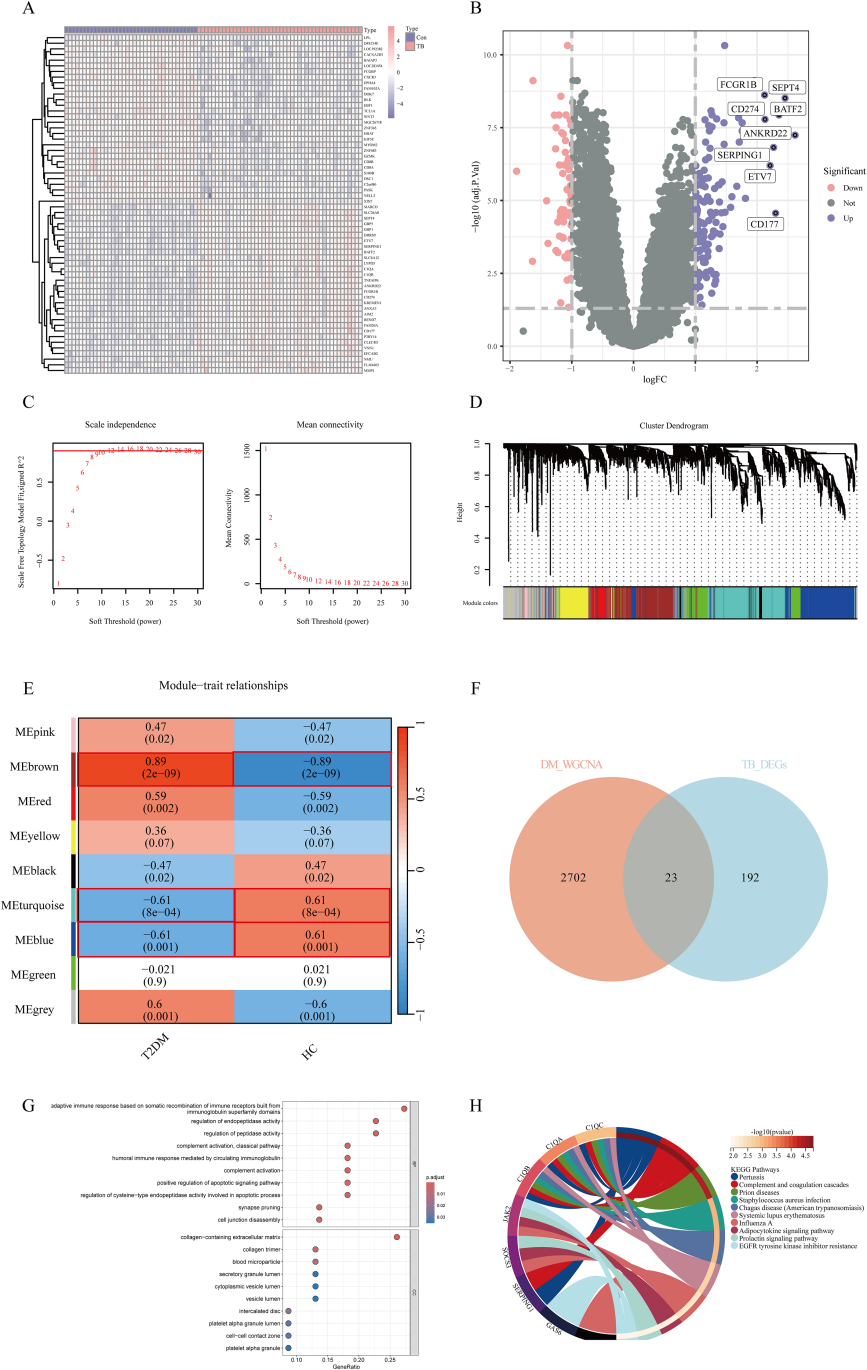
Identification and Pathway Enrichment of Shared Genes in TB and T2DM: **(A)** Heatmap of the top 60 DEGs in GSE28623. Pink grids represent upregulated genes, purple grids represent downregulated genes, and the color intensity indicates expression levels. **(B)** Volcano plot of DEGs in GSE28623. The x-axis represents log2 fold change (TB/Con), and the y-axis represents -log10(*P*-value). Pink and purple points represent significantly differentially expressed genes (pink: upregulated, purple: downregulated), while gray points represent non-significant genes. Con, control; TB, tuberculosis. **(C)** Soft-thresholding power plot for WGCNA analysis of key T2DM modules, showing scale-free topology. **(D)** Hierarchical clustering dendrogram of modules. **(E)** Correlation matrix between modules and traits. **(F)** Venn diagram showing the intersection of TB DEGs and T2DM key module genes. **(G)** GO enrichment analysis of shared genes. **(H)** KEGG pathway enrichment analysis of shared genes.

In the TB dataset GSE28623, a total of 192 DEGs were identified, including 131 upregulated DEGs and 61 downregulated DEGs. The heatmap (**Figure 1A**) displays the top 60 DEGs in TB, and the volcano plot (**Figure 1B**) illustrates the expression patterns of DEGs in TB.

### 3.2 Identification of key modules using WGCNA

Sample clustering was performed to detect outliers, and no samples were removed from the GSE166502 dataset. As shown in **Figure 1C**, the optimal soft-thresholding power value, which yields an R² value of approximately 0.9, indicates that the network closely approximates a scale-free topology. The mean connectivity plateaued as the adjacency function gradually approached zero. According to the dynamic tree cut algorithm, nine modules were identified in the co-expression network constructed from the T2DM samples (**Figure 1D**). The correlation between modules and sample traits was calculated, and the resulting correlation matrix is shown in **Figure 1E**. In the matrix, the vertical axis represents different modules, and the horizontal axis represents various traits. Each square indicates the correlation coefficient and statistical significance (*P*-value) between a module and a trait. Correlation analysis revealed that the brown module (cor = 0.89, *P* < 0.05), turquoise module (cor = -0.61, *P* < 0.05), and blue module (cor = -0.61, *P* < 0.05) exhibited the most significant correlations with T2DM.

### 3.3 Identification of shared genes and pathway enrichment

A total of 23 genes (*GAS6*, *SERPING1*, *PLSCR1*, *CYP1B1*, *CTNNAL1*, *ATF3*, *TNFSF10*, *ADM*, *S100A8*, *C1QB*, *BPGM*, *GADD45G*, *FGF13*, *MYOM2*, *JAK2*, *SOCS3*, *KCNJ2*, *CLEC2B*, *C1QA*, *CHPT1*, *KREMEN1*, *C1QC*, *CMBL*) were identified as Shared cross-talk genes between TB and T2DM based on the intersection of WGCNA modules and DEGs (**Figure 1F**). These 23 genes were further subjected to GO and KEGG enrichment analyses to explore shared regulatory pathways. The biological processes and cellular components are illustrated in **Figure 1G**; **Supplementary Table S1**. The biological processes encompass classical complement activation, humoral immune response mediated by immunoglobulin, synaptic pruning, complement activation, adaptive immune response based on somatic recombination of immune receptors built from immunoglobulin superfamily domains, cell junction disassembly, regulation of endopeptidase activity, positive regulation of apoptotic signaling pathway, regulation of peptidase activity, and regulation of cysteine-type endopeptidase activity involved in apoptotic processes. Cellular components include collagen-containing extracellular matrix, collagen trimer, blood microparticles, intercalated discs, platelet alpha granule lumen, cell-cell contact zone, platelet alpha granule, secretory granule lumen, cytoplasmic vesicle lumen, and vesicular lumen. The shared genes were primarily associated with the following pathways: pertussis, complement and coagulation cascades, prion diseases, Staphylococcus aureus infection, Chagas disease (American trypanosomiasis), systemic lupus erythematosus, influenza A, adipocytokine signaling pathway, prolactin signaling pathway, and EGFR tyrosine kinase inhibitor resistance (**Figure 1H**).

### 3.4 Machine learning for key gene identification

Four machine learning algorithms—LASSO regression, RF, SVM, and GMM—were employed to screen the 23 shared genes and identify potential key genes related to the mechanisms by which T2DM impacts TB.

LASSO regression identified 10 non-zero coefficients when the minimal mean squared error was selected at log (λ). These 10 genes were *MYOM2*, *GADD45G*, *ATF3*, *CMBL*, *CLEC2B*, *GAS6*, *CHPT1*, *C1QC*, *SERPING1*, and *CYP1B1* (**Supplementary Figures S2A, B**). The RF model achieved the highest accuracy when the number of selected genes was set to N=17 (**Supplementary Figure S2C**), identifying the following 17 genes: *GADD45G*, *KREMEN1*, *SERPING1*, *CHPT1*, *GAS6*, *CYP1B1*, *SOCS3*, *ATF3*, *JAK2*, *C1QC*, *CMBL*, *TNFSF10*, *BPGM*, *C1QB*, *KCNJ2*, *C1QA*, and *CTNNAL1.* Using the SVM-RFE method, the model reached the highest accuracy of 0.878 with the lowest cross-validation error of 0.122 when selecting 22 genes (**Supplementary Figures S2D, E**). The 22 selected genes were *GAS6*c, *SERPING1*, *PLSCR1*, *CYP1B1*, *CTNNAL1*, *ATF3*, *TNFSF10*, *ADM*, *S100A8*, *C1QB*, *GADD45G*, *FGF13*, *MYOM2*, *JAK2*, *SOCS3*, *KCNJ2*, *CLEC2B*, *C1QA*, *CHPT1*, *KREMEN1*, *C1QC*, and *CMBL.*

The intersection of the genes selected by the three algorithms resulted in 8 genes: *GADD45G*, *ATF3*, *CMBL*, *GAS6*, *CHPT1*, *C1QC*, *SERPING1*, and *CYP1B1* (**Supplementary Figure S2F**). Finally, the GMM model was employed to classify and refine the set of shared key genes, ultimately identifying the subset with the highest AUC combination: *GADD45G*, *GAS6*, *CHPT1*, *SERPING1*, and *CYP1B1* (**Supplementary Figure S2G**).

### 3.5 Expression levels of key genes and construction of predictive models

**Figure 2:**
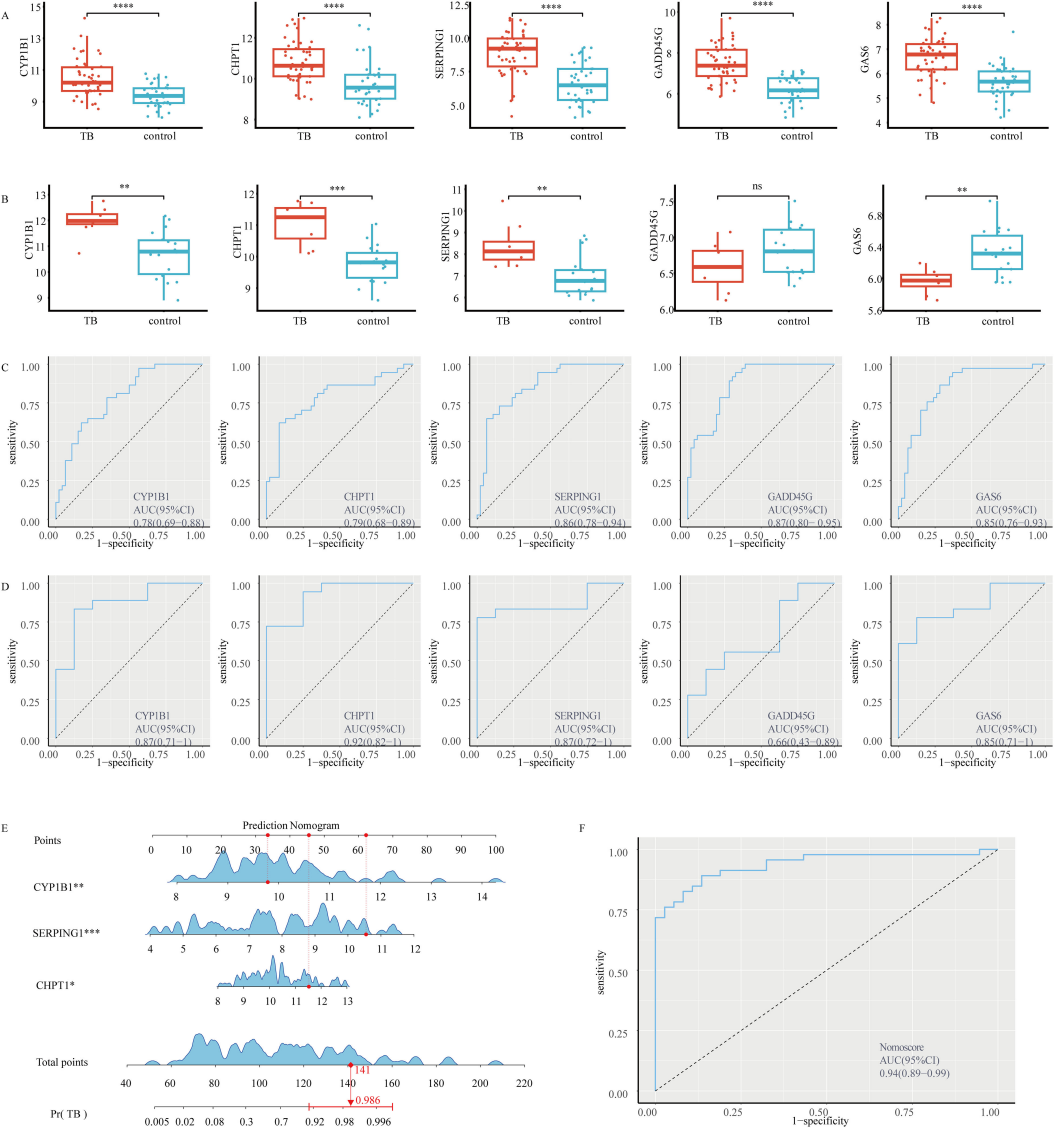
Relative expression levels, predictive performance, and nomogram construction of five key genes in the training and validation cohorts: **(A)** Relative expression levels of the five key genes in the test dataset GSE28623. **(B)** Relative expression levels of the five key genes in the validation dataset GSE34608. **(C)** ROC curves of the five key genes (*CHPT1*, *CYP1B1*, *GADD45G*, *GAS6*, and *SERPING1*) in the test dataset GSE28623. **(D)** ROC curves of the five key genes in the validation dataset GSE34608. **(E)** Nomogram model constructed based on cross-talk genes (*CYP1B1*, *SERPING1*, and *CHPT1*). **(F)** ROC curve of the nomogram. Statistical significance was denoted as follows: ns (not significant, *P* > 0.05), *P < 0.05, **P < 0.01, ***P < 0.001, and ****P < 0.0001.

The relative expression levels of the five key genes in the training and validation sets are shown in **Figures 2A, B**. Among these, *CHPT1*, *SERPING1*, and *CYP1B1* exhibited consistent expression patterns in both the training and validation sets. To assess the predictive ability of these five key genes for TB progression, ROC curves were constructed based on individual characteristics and gene expression levels. The AUC and corresponding 95% confidence intervals (CIs) for each gene were determined in both the test and validation datasets.

In the test dataset, the AUC values were as follows: *CHPT1* (AUC = 0.786, 95% CI: 0.682-0.890), *CYP1B1* (AUC = 0.784, 95% CI: 0.688-0.881), *GADD45G* (AUC = 0.874, 95% CI: 0.802-0.946), *GAS6* (AUC = 0.845, 95% CI: 0.758-0.931), *SERPING1* (AUC = 0.856, 95% CI: 0.775-0.937) (**Figure 2C**). For the GSE34608 validation dataset, the results were: *CHPT1* (AUC = 0.924, 95% CI: 0.818-1), *CYP1B1* (AUC = 0.868, 95% CI: 0.713-1), *GADD45G* (AUC = 0.66, 95% CI: 0.426-0.893), *GAS6* (AUC = 0.854, 95% CI: 0.707-1), *SERPING1* (AUC = 0.868, 95% CI: 0.724-1) (**Figure 2D**).

Based on the expression patterns and AUC values, three genes—*CHPT1*, *SERPING1*, and *CYP1B1*—were selected as cross-talk genes critical for TB progression in T2DM patients and used to construct a nomogram model. In the nomogram (**Figure 2E**), the relative expression levels of each gene were quantitatively marked, enabling the calculation of a total score to evaluate the risk of TB progression in individuals with T2DM. Moreover, the AUC for the nomogram was 0.938 (95% CI 0.885–0.990), highlighting the superior performance of the model (**Figure 2F**).

### 3.6 Immune infiltration analysis

**Figure 3:**
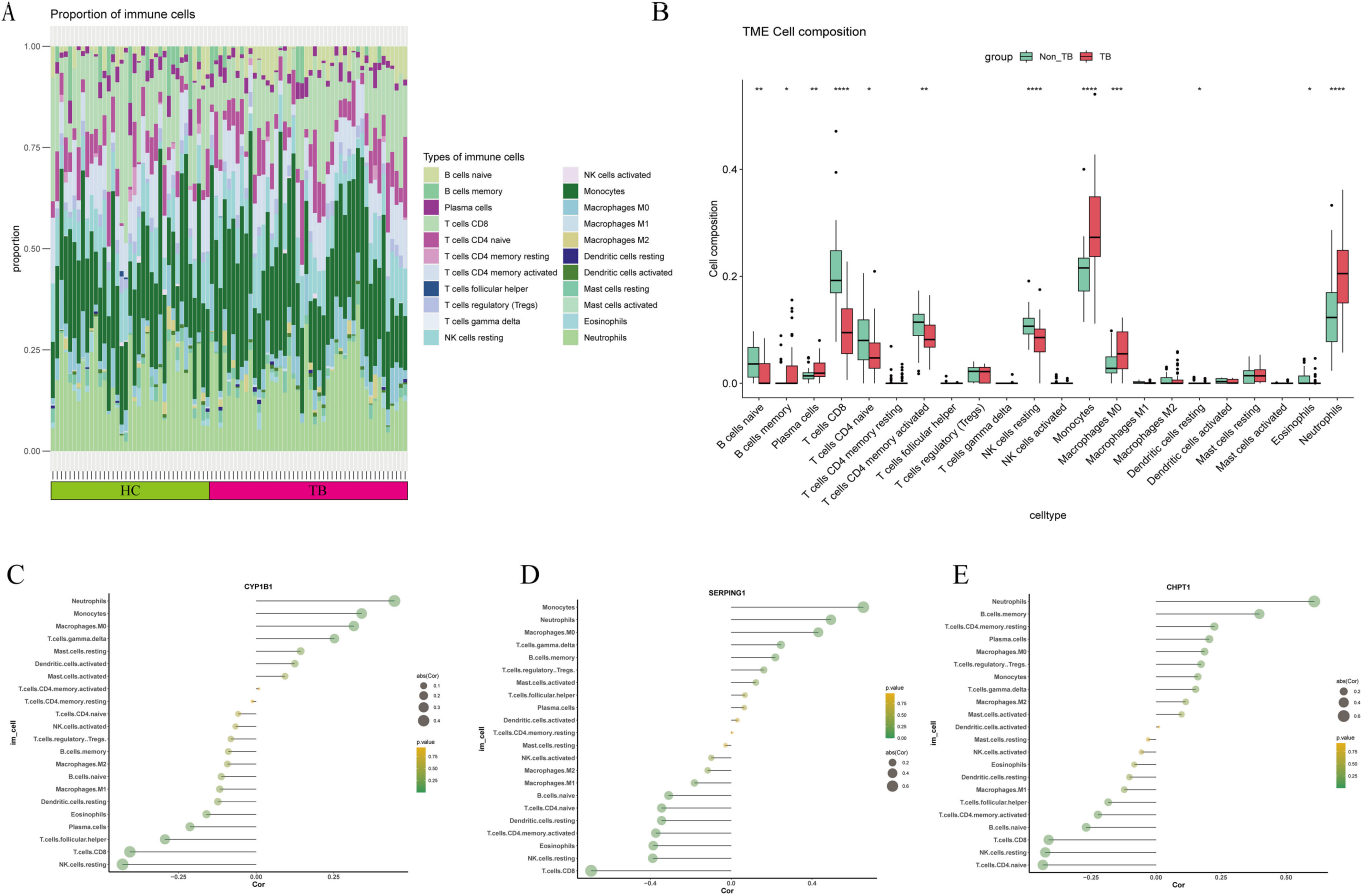
Immune Cell Infiltration Analysis: **(A)** Stacked plot showing the relative proportions of 24 immune cell types in different samples from the TB and control groups. **(B–D)** Correlation between the three key genes (*CYP1B1*, *SERPING1*, and *CHPT1*) and immune cells. **(E)** Boxplot comparing immune cell expression between the TB and control groups. Statistical significance was denoted as follows: ns (not significant, *P* > 0.05), *P < 0.05, **P < 0.01, ***P < 0.001, and ****P < 0.0001.

CIBERSORT was used to evaluate the proportion of 22 immune cell types in the TB dataset (**Figure 3A**). In the TB samples, significant upregulation was observed in the abundance of memory B cells, plasma cells, monocytes, macrophages (M0), and neutrophils. In contrast, downregulation was observed in naive B cells, CD8 T cells, naive CD4 T cells, activated memory CD4 T cells, resting NK cells, resting dendritic cells, and eosinophils (**Figure 3B**).

Further correlation analysis between the three key genes and the proportions of immune cells in the TB samples revealed interesting associations. *CYP1B1* demonstrated positive correlations with neutrophils, monocytes, macrophages (M0), and T cells gamma delta, while showing negative correlations with T cells follicular helper, CD8+ T cells, and resting NK cells. *SERPING1* was positively correlated with monocytes, neutrophils, macrophages (M0), T cells gamma delta, and B cells memory, while negatively correlated with B cells naive, CD4 T cells naive, dendritic cells resting, T cells CD4 memory activated, eosinophils, NK cells resting, and T cells CD8. *CHPT1* exhibited positive correlations with neutrophils, B cells memory, and T cells CD4 memory resting, and negative correlations with T cells CD4 memory activated, B cells naive, T cells CD8, NK cells resting, and T cells CD4 naive (**Figures 3C–E**).

### 3.7 qPCR detection of cross-talk genes expression levels

**Figure 4:**
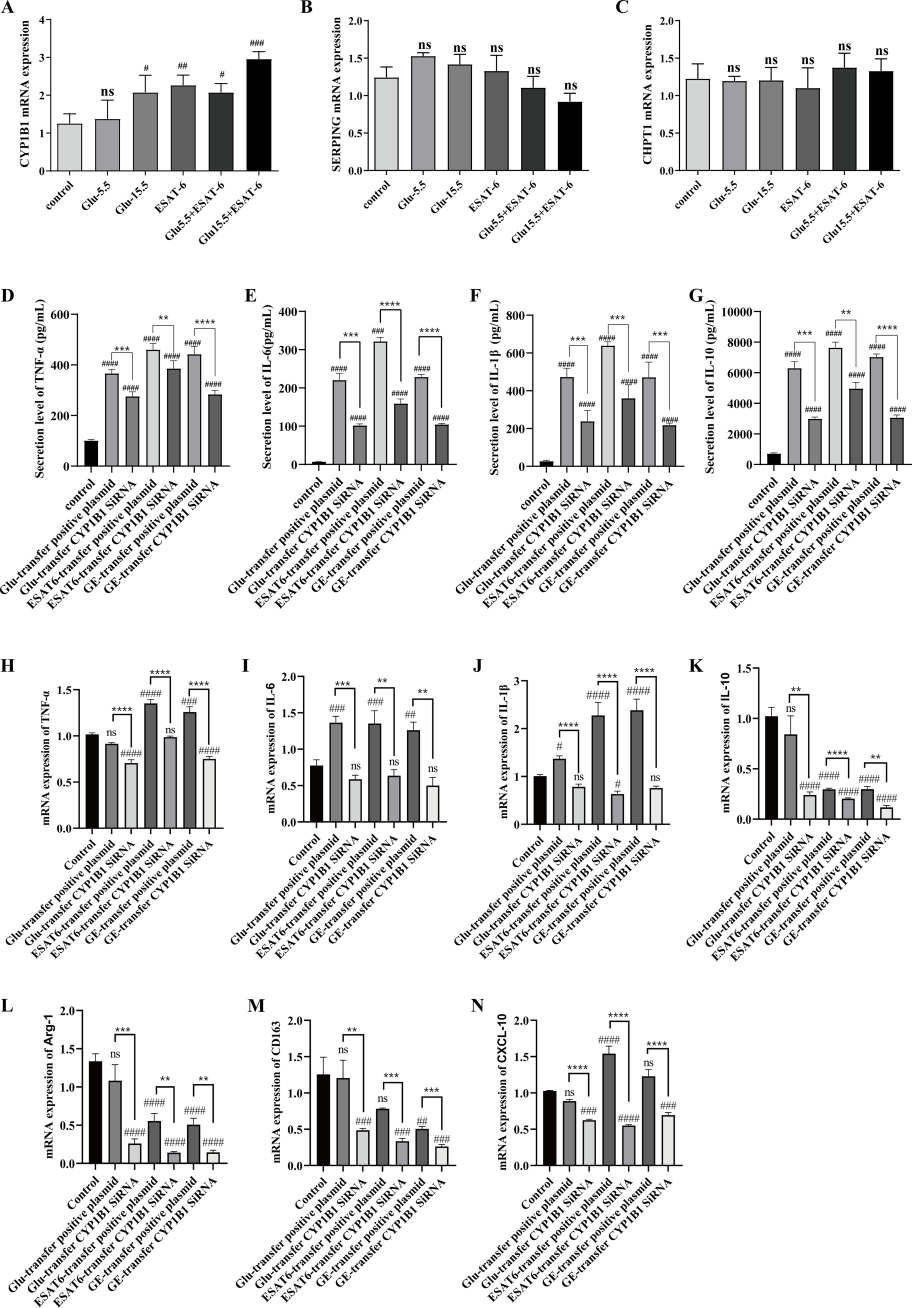
**(A)** Expression of *CYP1B1* in THP-1 cells under different treatments with various glucose levels and ESAT-6 stimulation. **(B)** Expression of *CHPT1* in THP-1 cells under different treatments. **(C)** Expression of *SERPING1* in THP-1 cells under different treatments. **(D–F)** ELISA analysis of pro-inflammatory cytokines TNF-α, IL-6, and IL-1β protein levels following *CYP1B1* siRNA transfection under different treatments. **(G)** ELISA analysis of IL-10 protein levels after *CYP1B1* knockdown. **(H–J)** qPCR analysis of mRNA expression levels of pro-inflammatory cytokines *TNF-α*, *IL-6*, and *IL-1β* after *CYP1B1* siRNA transfection. **(K)** qPCR analysis of *IL-10* mRNA expression following *CYP1B1* knockdown. **(L, M)** qPCR assay of the expression of M2 macrophage markers *CD163* and *Arg-1* after *CYP1B1* knockdown. **(N)** qPCR analysis of *CXCL10* expression following *CYP1B1* siRNA transfection. Control: Untreated control group; Glu-transfer positive plasmid: Positive plasmid transfection control + 15.5 μM glucose stimulation; Glu-transfer *CYP1B1* siRNA: *CYP1B1* siRNA transfection + 15.5 μM glucose stimulation; ESAT6-transfer positive plasmid: Positive plasmid transfection control + ESAT6 stimulation; ESAT6-transfer *CYP1B1* siRNA: *CYP1B1* siRNA transfection + ESAT6 stimulation; GE-transfer positive plasmid: Positive plasmid transfection control + 15.5 μM glucose + ESAT6 co-stimulation; GE-transfer *CYP1B1* siRNA: *CYP1B1* siRNA transfection + 15.5 μM glucose + ESAT6 co-stimulation. Statistical significance is denoted as follows: ns, non-significant (*P* > 0.05); ^#^ *P* < 0.05, ^##^ *P* < 0.01, ^###^ *P* < 0.001, ^####^ *P* < 0.0001 (vs. control); *P < 0.05, **P < 0.01, ***P < 0.001, ****P < 0.0001 (vs. transfer positive plasmid).

Given the pivotal role of macrophages in the immune responses to both T2DM and TB, we employed a human acute monocytic leukemia cell (THP-1) model to investigate the regulatory effects of the cross-talk genes *CHPT1*, *SERPING1*, and *CYP1B1* in inflammatory responses. To simulate the distinct pathological conditions of T2DM and TB, cells were subjected to six treatment regimens: Control (unstimulated), 15.5 μM glucose (Glu-15.5), 5.5 μM glucose (Glu-5.5), ESAT-6, 15.5 μM glucose + ESAT-6 (Glu-15.5+ ESAT-6), and 5.5 μM glucose + ESAT-6 (Glu-5.5+ ESAT-6). The results revealed significant differences in *CYP1B1* expression across the treatment groups, as detailed below:

Compared to the control group, *CYP1B1* mRNA expression in the Glu-5.5 group showed no significant change, indicating that physiological glucose concentrations do not significantly modulate *CYP1B1* expression. In contrast, *CYP1B1* mRNA expression was significantly upregulated in the Glu-15.5 group (*P* < 0.05), suggesting that a high glucose state activates *CYP1B1* expression. Under ESAT-6 treatment, *CYP1B1* expression also significantly increased (*P* < 0.01). The combination of 5.5 μM glucose and ESAT-6 further upregulated *CYP1B1* expression (*P* < 0.05). The highest expression of *CYP1B1* was observed under the combined treatment of 15.5 μM glucose and ESAT-6 (*P* < 0.001) (**Figure 4A**). This significant upregulation suggests that *CYP1B1* may serve vital functions in the co-existing pathological conditions of T2DM and TB. No significant differences in gene expression were observed for *SERPING1* and *CHPT1* under the aforementioned treatment conditions (**Figures 4B, C**).

### 3.8 Transfection of CYP1B1 siRNA reduced CYP1B1 expression

The function of *CYP1B1* in the comorbidity of T2DM and TB was further investigated by knocking down its expression in macrophages through siRNA transfection and observing the changes in cytokines and key pathway molecules before and after transfection.

The qPCR results demonstrated that compared to the negative control and the plasmid transfection group (Si-NC), the *CYP1B1* siRNA transfection group exhibited significantly reduced mRNA expression levels (*P* < 0.05). No statistically significant difference was observed between the *CYP1B1* siRNA group and the GAPDH plasmid transfection group (Si-GAPDH) (*P* > 0.05) (**Supplementary Figure S3A**). Thus, the siRNA, along with its transfection concentration and duration in this experiment, effectively silenced *CYP1B1* expression, highlighting its suitability for subsequent experiments.

Western blot analysis further confirmed a significant decrease in CYP1B1 protein levels. Compared with the Si-NC group, *CYP1B1* siRNA transfection markedly suppressed *CYP1B1* protein expression (*P* < 0.05). These results demonstrated that the reduced mRNA levels directly led to decreased protein expression. No significant difference was detected in the protein expression levels between the *CYP1B1* siRNA and Si-GAPDH groups (*P* > 0.05) (**Supplementary Figures S3B, C**). The WB results were consistent with qPCR data, confirming transfection efficacy.

### 3.9 CYP1B1 expression is associated with inflammatory responses and immune regulation

Following *CYP1B1* siRNA transfection, we further evaluated the impact of *CYP1B1* knockdown on various inflammatory and immune regulatory factors. The protein and mRNA levels of pro-inflammatory cytokines, e.g., *TNF-α*, *IL-6*, and *IL-1β*, and the anti-inflammatory cytokine *IL-10* were analyzed using ELISA and qPCR. Additionally, the mRNA expression levels of characteristic macrophage polarization markers, e.g., *CD163* and *Arg-1*, and the chemokine *CXCL10* were assessed.

After *CYP1B1* siRNA transfection, the protein and mRNA expression levels of key inflammatory mediators decreased significantly. According to the ELISA and qPCR measurements, the pro-inflammatory cytokines *TNF-α*, *IL-6*, and *IL-1β* showed decreased expression across the treatment groups with 15.5 μM glucose, ESAT-6, and their combination, with the most notable suppression observed under combined stimulation (*P* < 0.01, **Figures 4D–F, H–J**). Similarly, the anti-inflammatory cytokine *IL-10* demonstrated reduced expression at both protein and transcript levels (**Figures 4G, K**). These results demonstrate a significant positive correlation between the expression levels of the above inflammatory factors and *CYP1B1* expression.

As detailed in **Figures 4L–N**, the expression of macrophage polarization markers *CD163* and *Arg-1* paralleled these findings, showing significant downregulation following CYP1B1 knockdown (*P* < 0.05). The chemokine *CXCL10* also exhibited comparable reductions (*P* < 0.05).

### 3.10 Single-sample gene set enrichment analysis

**Figure 5:**
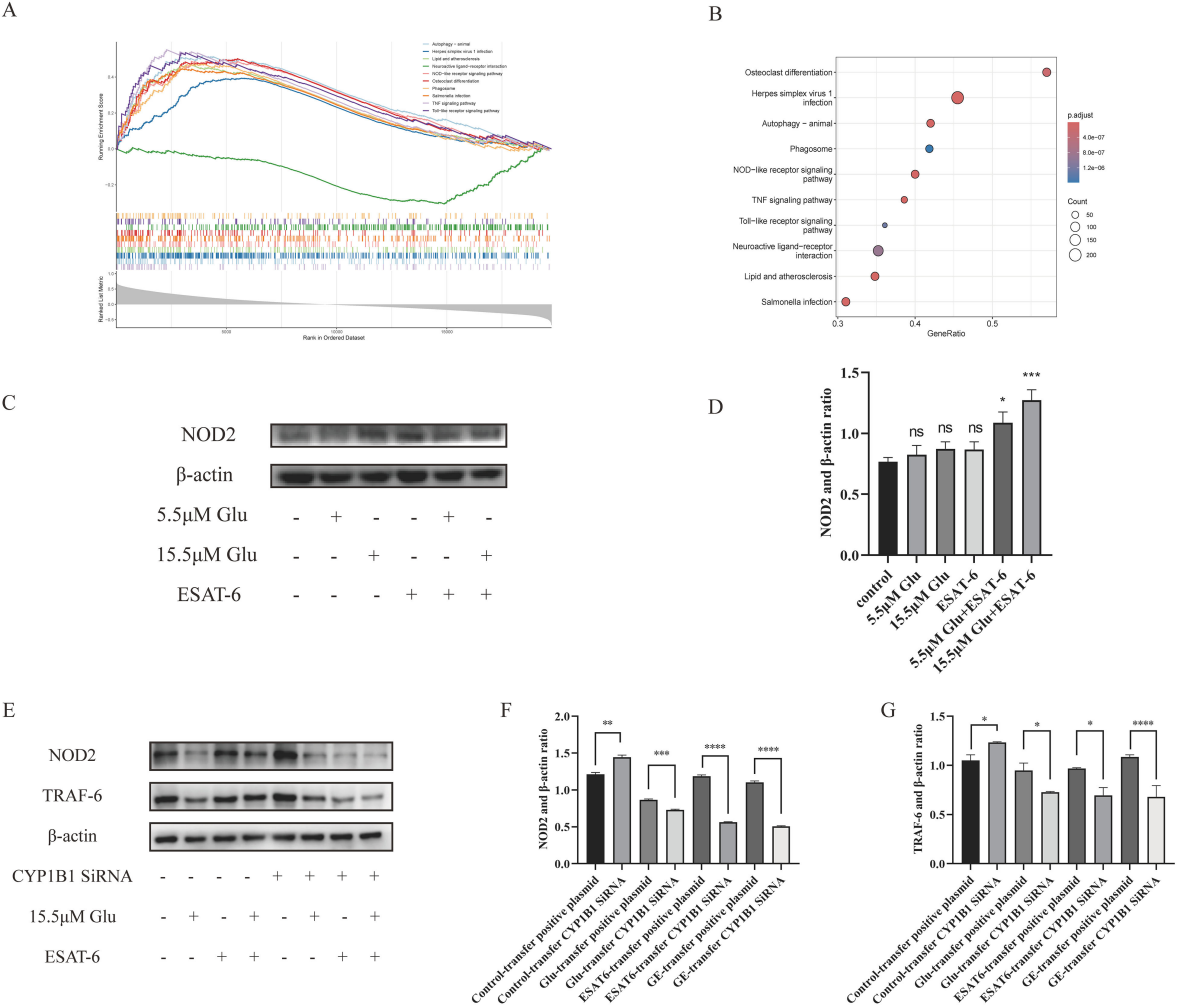
*CYP1B1* regulation of NOD2 and TRAF6 signaling in macrophages under various treatments: **(A, B)** KEGG pathway enrichment analysis showing the top ten pathways associated with TB progression. **(C, D)** Western blot analysis of NOD2 protein expression under different treatments. **(E–G)** Western blot analysis of NOD2 and TRAF6 expression following *CYP1B1* knockdown using siRNA transfection under various treatments. Statistical significance is denoted as follows: ns, not significant (*P* > 0.05), *P < 0.05, **P < 0.01, ***P < 0.001, and ****P < 0.0001. The control lane shown in panel **(E)** was derived from the same experimental blot. Reusing this control data allows for direct comparison across experiments under identical conditions. The polyclonal anti-NOD2 antibody may produce non-specific bands due to its broad reactivity. The band of interest (indicated by the arrow) was selected based on its expected molecular weight (~110 kDa).

*M. tuberculosis* infection triggers complex immune responses, particularly in the context of T2DM, and the altered immune responses may influence the clinical manifestation of TB. We performed ssGSEA to further investigate the potential KEGG pathways involved in TB progression, with a focus on the role of *CYP1B1*. The ten most significant pathways are presented in **Figures 5A, B**. The results revealed that *CYP1B1* is involved in multiple pathways, such as autophagy, NOD-like receptor signaling, phagosome formation, TNF signaling, and Toll-like receptor signaling.

### 3.11 Western blot validation of NOD2 and TRAF-6 signaling pathways

The ssGSEA results indicated that *CYP1B1* is significantly associated with the NOD-like receptor signaling pathway and the TNF signaling pathway. Preliminary experiments on the expression of NOD2 in macrophages found that the upregulation of *CYP1B1* might be related to NOD2 activation. Western blot analysis of NOD2 protein expression levels across different treatment groups revealed the following results. Compared to the control group, NOD2 protein expression was significantly upregulated in the 5.5 μM glucose + ESAT-6 and 15.5 μM glucose + ESAT-6 treatment groups (*P* < 0.05, **Figures 5C, D**). In the 15.5 μM glucose + ESAT-6 treatment group, the synergistic effects of a high glucose level and TB infection significantly activated NOD2 expression, resulting in the highest level of NOD2 protein expression.

TRAF6, a downstream adapter protein of NOD2, is critical in initiating downstream immune responses. Considering the enrichment results of the TNF signaling pathway and the close relationship between the NOD2 signaling pathway and TRAF6, we further investigated the changes in NOD2 and TRAF6 expression after *CYP1B1* siRNA transfection. Compared to the control group with siRNA transfection, the protein expression levels of both NOD2 and TRAF6 were elevated in the *CYP1B1* siRNA transfection group (*P* < 0.05). In the treatment group with 15.5 μM glucose, the expression of NOD2 and TRAF6 was significantly reduced compared to the group with *CYP1B1* siRNA transfection (*P* < 0.05). In the ESAT-6 treatment group, knocking down *CYP1B1* led to a significant reduction in the expression of both NOD2 and TRAF6 (*P* < 0.05). Under the combined treatment of 15.5 μM glucose + ESAT-6, the expression of NOD2 and TRAF6 was markedly decreased (*P* < 0.0001) (**Figures 5E–G**). These findings suggest that *CYP1B1* regulates the expression of NOD2 and TRAF6 in response to the dual stress of high glucose and *M. tuberculosis* infection.

## 4 Discussion

This study systematically unveils the potential molecular mechanisms underlying the TB-T2DM comorbidity, particularly the key regulator *CYP1B1* in the inflammatory response of this comorbidity.

Bioinformatics methods like DEGs and WGCNA were employed to identify multiple potential key genes and their associated modules. Further refinement using machine learning methods revealed the cross-talk genes *CYP1B1*, *SERPING1*, and *CHPT1*, which are closely related to the inflammatory response in TB-T2DM comorbidity.

Differential gene expression analysis identified 192 DEGs in TB patients. WGCNA revealed three modules significantly associated with T2DM. A comparison between the DEGs in TB patients and the key module genes associated with T2DM identified 23 shared genes. Functional enrichment analysis provided the biological context for these shared genes. GO analysis indicated that the shared genes were enriched in biological processes like complement activation and immunoglobulin-mediated humoral immune responses. KEGG pathway analysis highlighted key pathways like the complement and coagulation cascades and circulating immunoglobulin-mediated humoral immune responses.

The significant enrichment of complement activation, immunoglobulin-mediated humoral immune responses, and the complement-coagulation cascade in TB-T2DM comorbidity demonstrated their key functions in inflammatory imbalance and immune dysfunction. Excessive complement activation may serve as a key driver of TB and T2DM progression. The high-glucose environment of T2DM may induce abnormal expression of coagulation factors and complement components, potentially amplifying inflammatory responses and exacerbating microvascular complications (39). The activation of the coagulation system in TB patients is closely associated with the formation of tuberculous granulomas and tissue necrosis. Excessive activation of the complement system may exacerbate pulmonary damage by inducing localized coagulation (40). Declined immunoglobulin function may further weaken the host’s immune clearance capacity. Immunoglobulins serve as essential effector molecules in B cell-mediated humoral immunity, facilitating infection combating by binding to pathogen surface antigens, activating the complement system, and promoting pathogen clearance. In diabetic patients, the high-glucose environment may impair B cell function and alter the glycosylation of immunoglobulins, thereby reducing their effector functions (41). Specific immunoglobulins, such as IgM, are vital in the early clearance of *M. tuberculosis* and the regulation of late-stage disease progression (42). In TB-T2DM comorbidity, dysregulation of humoral immunity may further impair the host’s ability to clear *M. tuberculosis*, thus prolonging the infection and increasing the disease burden.

The four machine learning methods, expression pattern analysis, and ROC curve evaluation across multiple dimensions identified three key genes from the 23 shared genes: *CYP1B1*, *SERPING1*, and *CHPT1*. Pathway enrichment analysis of the three genes revealed their potential roles in immune responses, inflammation, lipid metabolism, cell growth, and immune evasion in both T2DM and TB. Immune infiltration analysis revealed the cellular-level immunopathological characteristics of TB-T2DM comorbidity. In TB patients, the neutrophils, monocytes, and M0 macrophages increase significantly, while the CD8+ T cells and natural killer (NK) cells decrease markedly. Neutrophils protect the host via the oxidative killing of mycobacteria and serve as critical innate immune cells for combating TB infection (43), and elevated absolute neutrophil counts are a hallmark of TB-T2DM (44). Following initial *M. tuberculosis* infection, monocytes rapidly migrate to the lungs and differentiate into macrophages and dendritic cells for antigen presentation and cytokine secretion (45). The significant increase in M0 macrophages may result from the *M. tuberculosis* infection-induced shift toward the M0 phenotype, enabling the bacteria to evade host immune clearance (46). CD8+ T cells also produce pro-inflammatory cytokines, including type 1 and type 17 cytokines in TB (47). Upon stimulation by *M. tuberculosis* antigens, CD8+ T cells exhibit increased expression of type 1 (IFN-γ and IL-2) and type 17 (IL-17F) cytokines, but their expression of cytotoxic markers such as perforin, granzyme B, and CD107a is significantly reduced. These findings suggest a critical link between CD8+ T cells and the pathogenesis of TB-T2DM comorbidity (48). NK cells are effector cells of innate immunity, serving vital functions in early infection by activating phagocytes at the infection site. The production of IFN-γ, IL-17, and IL-22 by NK cells is crucial for the host’s defense against *M. tuberculosis*. Studies indicated that *M. tuberculosis* suppresses NK cell activation receptors and reduces cytokine production, leading to decreased NK cells or impaired function during TB infection (49). Interestingly, *CYP1B1* expression positively correlated with the infiltration levels of neutrophils, monocytes, and M0 macrophages, while negatively correlating with CD8+ T cells and NK cells. Thus, *CYP1B1* may regulate the activity of monocytes and neutrophils, thereby enhancing innate immune responses while suppressing adaptive immune functions. This dual regulatory function of *CYP1B1* in immune modulation may explain the heightened inflammatory responses and reduced anti-infective capacity in diabetic patients with TB infection.

The experimental validation focused on the expression of the cross-talk genes *CYP1B1*, *CHPT1*, and *SERPING1* in *in vitro* models of T2DM-TB comorbidity as well as their impact on inflammatory responses and immune regulation. The results showed that *CYP1B1* was significantly upregulated in response to the combined stimulation of high glucose and ESAT-6. Further siRNA transfection successfully knocked down *CYP1B1* expression, leading to a significant reduction in inflammatory cytokines. Thus, *CYP1B1* may influence the intensity of inflammatory responses by regulating the activity of immune cells. Silencing *CYP1B1* also resulted in decreased expression of M2 macrophage markers CD163 and Arg-1, indicating its potential involvement in the anti-inflammatory polarization of macrophages. Further studies revealed that knocking down *CYP1B1* significantly affected the expression of NOD2 and TRAF-6. Stimulated by high glucose levels and ESAT-6, the protein levels of NOD2 and TRAF-6 were markedly reduced, suggesting the crucial function of *CYP1B1* in regulating the inflammatory response of the TB-T2DM comorbidity via the modulation of NOD2 and TRAF-6.

*CYP1B1* is a member of the cytochrome P450 superfamily and is primarily involved in the metabolism of fat-soluble substances such as fatty acids and steroids (50, 51), particularly omega-3 and omega-6 fatty acids (52). Such fatty acids are fundamental for constructing cell membranes and regulating lipid signaling pathways. As a result, *CYP1B1* may influence cell membrane fluidity, immune cell function, and the intensity of immune responses by modulating lipid metabolism (53). In T2DM-TB comorbidity, the dysregulation of lipid metabolism is often accompanied by heightened inflammatory responses (54). In T2DM, lipid metabolism imbalance is a key manifestation of insulin resistance and chronic low-grade inflammation (55). High-fat diets and obesity can trigger inflammatory responses in adipose tissue, which affect the systemic immune environment through the secretion of adipokines (e.g., adipocytokines and pro-inflammatory cytokines), thereby exacerbating immune dysregulation in diabetic patients (56). Studies have reported that with high-fat diet-induced obesity, mice lacking *CYP1B1* were leaner and more resistant to obesity than the control (57). Meanwhile, studies have linked CYP121, CYP125, and CYP128 closely to the growth and survival of *M. tuberculosis*. High-throughput screening and fragment-based drug discovery methods have identified selective CYP inhibitors, thus providing a foundation for developing novel anti-tuberculosis drugs (58). The lipid metabolism disorders in TB patients also impair the immune function of macrophages, which typically clear pathogens through phagocytosis after *M. tuberculosis* infection (59). In T2DM patients, the metabolic reprogramming of fatty acids may lead to macrophage dysfunction, reducing their ability to clear *M. tuberculosis (* 60). *CYP1B1* may exacerbate immune dysregulation by affecting the lipid metabolism pathways, further delaying the immune response to TB in diabetic patients (61).

SERPING1, in particular, is important in the complement system (62). The complement system is a vital component of the immune system, protecting against infections by lysing bacteria, clearing immune complexes, and regulating immune responses (63). Research reported that the impaired monocyte function in diabetic patients may weaken their role in *M. tuberculosis* infection. The reduced binding capacity of monocytes to *M. tuberculosis* in diabetic individuals may be linked to the altered complement system. SERPING1 overexpression could lead to abnormal complement system activation, triggering excessive inflammation and immune responses, thereby promoting immune evasion by *M. tuberculosis (* 64). However, research on its specific function in the shared pathological mechanisms of T2DM and TB remains insufficient, warranting further exploration. CHPT1 is a member of the choline phosphotransferase family and primarily catalyzes the synthesis of phosphatidylcholine, which is crucial for maintaining the structure and function of cell membranes. By promoting phosphatidylcholine production, CHPT1 contributes to cell membrane renewal and stability (65). Blocking choline uptake with pharmacological inhibitors and CTL1-specific antibodies was found to alter cytokine secretion patterns, with increased levels of TNF-α and IL-6 but decreased levels of IL-10 (66). In TB-T2DM comorbidity, phosphatidylcholine synthesis may be important in processes such as cell interactions and cytokine release during inflammatory responses (67). The *in vitro* models in this study exhibited no statistically significant differences in the expression levels of *CHPT1* and *SERPING1* across treatments compared to controls. *CHPT1* is primarily associated with lipid metabolism and cell membrane modifications, which may be upregulated in adipocytes. *SERPING1* is mainly involved in complement system activation, likely requiring complement participation or a more complex *in vivo* immune microenvironment to fully manifest its regulatory effects. However, this does not imply their irrelevance in the pathogenesis of T2DM and TB. On the contrary, they may play indirect or synergistic roles in complex biological systems. Thus, future studies may modify the experimental conditions or construct more sophisticated animal models to reach definitive conclusions.

siRNA transfection effectively suppressed *CYP1B1* expression, leading to significant downregulation of both pro-inflammatory and anti-inflammatory cytokines. These results establish a positive correlation between *CYP1B1* expression and the production of inflammation-related factors. Silencing *CYP1B1* also affected the expression of M2 macrophage markers (CD163 and Arg-1). CD163 is a prominent marker of M2 macrophages, typically associated with anti-inflammatory and tissue repair functions. It reduces oxidative stress and inflammatory responses by clearing hemoglobin-haptoglobin complexes (68). Arg-1 is another key marker of M2 macrophages, which converts arginine into ornithine and polyamines to promote cell proliferation and tissue repair (69). Knocking down *CYP1B1* significantly reduced the expression of CD163 and Arg-1 in TB-T2DM comorbidity. Thus, *CYP1B1* may influence the anti-inflammatory polarization of macrophages by regulating the expression of CD163 and Arg-1, thereby exacerbating inflammatory responses. CXCL10 is a chemokine that, upon binding to the CXCR3 receptor, participates in immune cell recruitment and inflammatory response regulation. Its recruitment of Th1 cells and NK cells to sites of inflammation enhances immune reactions (70). The experimental results also showed significantly decreased mRNA expression of CXCL10 after *CYP1B1* knockdown, indicating that the absence of *CYP1B1* in TB-T2DM comorbidity suppresses immune cell recruitment. In summary, CYP1B1 knockdown reduced both M1 and M2 macrophage polarization. Bioinformatics analysis of immune infiltration revealed that TB patients exhibited upregulated M0 macrophages, while their M1/M2 polarization remained unchanged compared to healthy controls. Therefore, while *M. tuberculosis* alters host metabolism, the metabolic reprogramming is not affected. Likewise, our experimental results demonstrated that although *CYP1B1* influences the metabolism, it does not affect the reprogramming in macrophages.

The above research found that silencing *CYP1B1* reduces the polarization of both M1 and M2 macrophages, indicating the significant involvement of *CYP1B1* in the immune function of macrophages. The underlying mechanisms are explored based mainly on the ssGSEA results of *CYP1B1* in bioinformatics analysis, particularly the NOD-like receptor and TNF signaling pathways. NOD2 is a crucial pattern recognition receptor involved in sensing pathogens and activating immune responses (71). RIP2-mediated ubiquitination of NOD2 interacts with TRAF6, thus activating the NF-κB signaling pathway and initiating downstream immune responses (72). This pathway is key to immune cell activation, inflammatory response regulation, and immune evasion in TB (73). Additionally, TRAF6, as an E3 ubiquitin ligase, mediates the polyubiquitination of itself and downstream signaling molecules, further enhancing inflammatory responses (74). Our experimental results showed significantly downregulated expression of NOD2 and TRAF6 after silencing *CYP1B1*, suggesting that they may be regulated by *CYP1B1*, which is consistent with the ssGSEA results from bioinformatics analysis. However, in the control group with *CYP1B1* siRNA transfection, the protein expression levels of NOD2 and TRAF6 showed an increasing trend relative to the control group with siRNA transfection. A possible explanation is that with *CYP1B1* knockdown, cells may activate other inflammatory pathways to compensate for the resultant metabolic stress. As *CYP1B1* knockdown leads to intracellular metabolic disturbances, NOD2, as an important pattern recognition receptor, may be upregulated to enhance immune responses. Similarly, the basal expression level of TRAF6 significantly increased after *CYP1B1* knockdown. TRAF6 is vital in inflammatory signaling, and its upregulation may be related to the activation of the NOD2 pathway, further indicating that cells maintain downstream signaling and inflammatory responses by increasing TRAF6 expression after *CYP1B1* knockdown.

**Figure 6:**
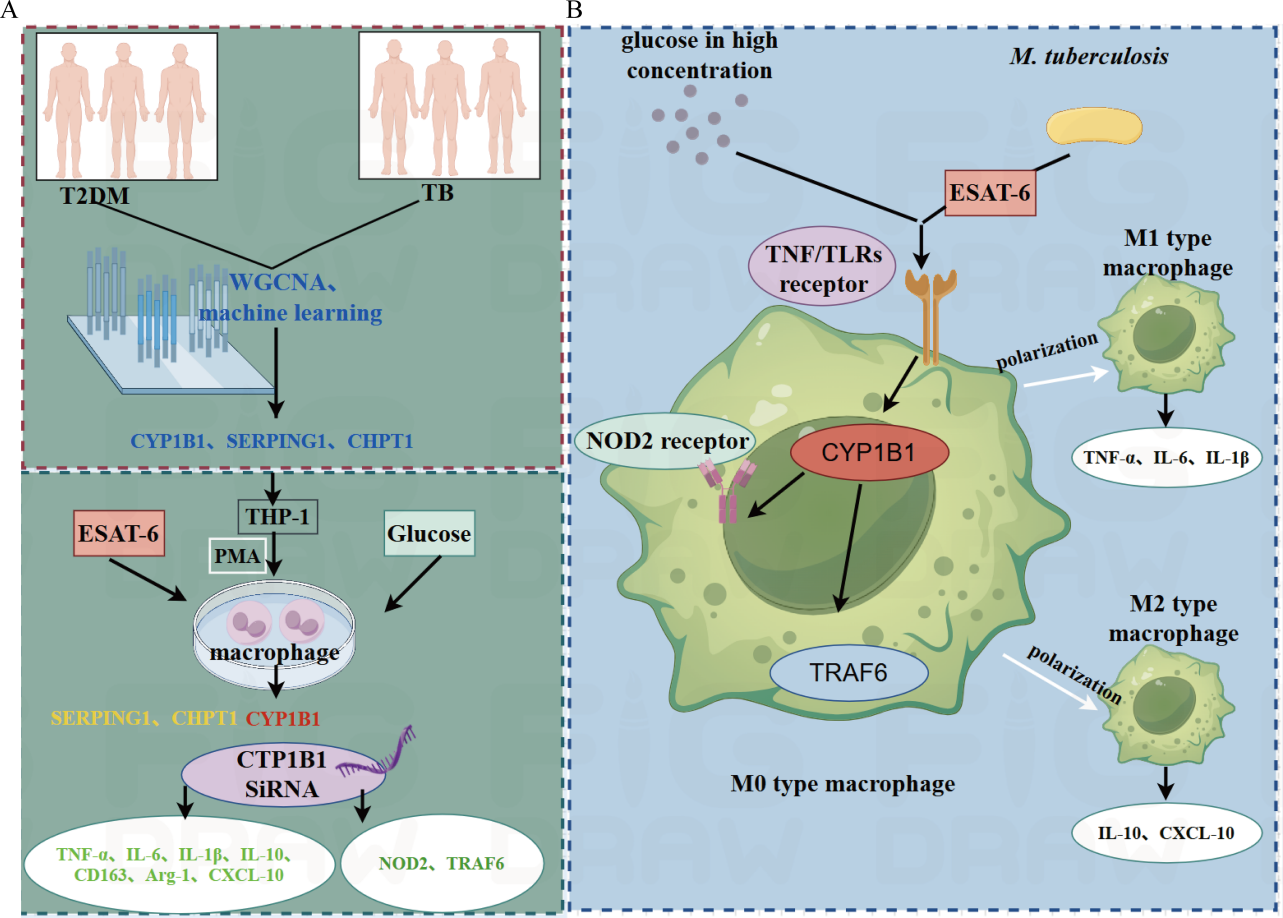
Discussion diagram: **(A)** The left portion (green section) summarizes the experimental results of this study: Transcriptome data from T2DM and TB patients were analyzed using WGCNA and machine learning methods, identifying *CYP1B1*, *SERPING1*, and *CHPT1* through enrichment analysis. As immune infiltration results exhibited an association with M0 macrophages, this study employed THP-1 M0 macrophages for experiments. However, only *CYP1B1* mRNA levels were upregulated in THP-1 macrophages (highlighted in red), while the other two genes remained unchanged (highlighted in yellow). Further experiments using *CYP1B1* siRNA demonstrated downregulation of inflammation-related factors and receptors (highlighted in green). **(B)** The right portion (blue section) presents hypotheses based on experimental findings: High glucose concentrations and *M. tuberculosis*-secreted ESAT-6 stimulate TNF and Toll-like receptors (TLRs). The resulting cascade signals are regulated by *CYP1B1*, subsequently transmitting to NOD2 receptors and TRAF6, ultimately affecting macrophage autophagy. Additionally, these signals induce polarization of M0 macrophages, which differentiate into M1 macrophages that secrete TNF-α, IL-6, and IL-1β while activating CD163 and Arg-1 to polarize into M2 macrophages that secrete IL-10 and CXCL-10.

Based on the above findings, we reasonably speculate that *CYP1B1* may be a key regulator in the inflammatory response of TB-T2DM comorbidity through the following mechanisms. The reasoning process is illustrated in **Figure 6**.

1. *CYP1B1* enhances inflammatory responses via the NOD2-TRAF6-NF-κB pathway: *CYP1B1* may regulate NOD2 and TRAF6, promoting NF-κB activation and amplifying immune responses triggered by TB infection. The high-glucose environment may further induce *CYP1B1* expression, exacerbating NF-κB activation and causing excessive inflammatory responses and tissue damage.
2. *CYP1B1* disrupts the dynamic balance between pro-inflammatory and anti-inflammatory responses: Silencing *CYP1B1* reduces the expression of both pro-inflammatory cytokines (TNF-α, IL-6, and IL-1β) and anti-inflammatory cytokines (IL-10). Meanwhile, the expression of M2 macrophage markers (CD163, Arg-1) and the chemokine CXCL-10 is suppressed. This imbalanced immune regulation may increase the susceptibility of diabetic patients to TB infection and exacerbate the severity of inflammatory responses.
3. Metabolic dysregulation affects immune function modulation: *CYP1B1* participates in lipid metabolism, influencing fatty acid metabolism and cell membrane fluidity and, in turn, regulating immune cell function. In TB-T2DM comorbidity, *CYP1B1*-induced lipid metabolism dysregulation may impair the ability of macrophages to clear *M. tuberculosis*, creating a vicious cycle of inflammation and metabolic abnormalities.

A limitation of this study is that *in vitro* models may not fully reflect the complex inflammatory and immune regulatory mechanisms *in vivo*. T2DM is a chronic process, and which is a risk factor for Mycobacterium tuberculosis infection (6). Although ESAT-6/glucose disease models were used in the researches, however, treated glucose or ESAT-6 in macrophage does not fully reflect the inflammation entire process of for T2DM or Mycobacterium tuberculosis infection. Nevertheless, the mechanism found in this study may reflect an early response via macrophage. In the future study, we will utilize diabetes model mouse and live Mycobacterium tuberculosis, e.g. Ra or Rv strain to explore the further mechanism following this research results.

## 5 Conclusion

This study identified *CYP1B1*, *SERPING1*, and *CHPT1* as cross-talk genes between TB and T2DM. Experimental results showed that *CYP1B1* was significantly upregulated in response to the combined stimulation of high glucose concentration and ESAT-6. *CYP1B1* knockdown markedly reduced the expression of inflammatory cytokines (TNF-α, IL-6, IL-1β, and IL-10), M2 macrophage polarization markers (CD163 and Arg-1), and chemokine CXCL-10 and suppressed the expression of NOD2 and TRAF6. These findings demonstrate that in the macrophage inflammatory responses mediated by ESAT-6, high glucose, or combined stimulation, the expression of NOD2, TRAF6, and cytokines positively correlates with *CYP1B1* levels, indicating *CYP1B1* as a crucial regulator in TB-T2DM inflammation by modulating inflammatory factors and NOD2/TRAF6 expression.

## Data Availability

Publicly available datasets were analyzed in this study. This data can be found here: https://www.ncbi.nlm.nih.gov/geo/.

## Supplementary material

Supplementary Figure 1 Flow chart of this research.

Supplementary Figure 2 Machine learning algorithms identifying key genes related to the mechanisms by which T2DM affects TB. **(A)** LASSO coefficient distribution plot. **(B)** LASSO coefficient profile plot, where the vertical dashed lines indicate the log(λ) values corresponding to the minimum mean squared error and one standard error away from the minimum mean squared error. The plot also shows the number of variables with non-zero coefficients in LASSO regression. **(C)** RF model achieving the highest accuracy at N=17. **(D, E)** SVM-RFE feature: 5-fold cross-validation accuracy plot and error plot. **(F)** Venn diagram showing the intersection of genes selected by LASSO, RF, and SVM-RFE. **(G)** Gaussian mixture model determining the logistic regression model pattern related to AUC values.

Supplementary Figure 3
**(A)** qPCR analysis of *CYP1B1* mRNA expression following transfection with *CYP1B1* SiRNA. **(B, C)** Western blot analysis showing the reduction of *CYP1B1* protein expression after SiRNA-mediated knockdown. Note: Statistical significance is denoted as follows: ns, not significant (*P* > 0.05), **P* < 0.05, ***P* < 0.01, and ****P* < 0.001. The polyclonal anti-CYP1B1 antibody may produce non-specific bands due to its broad reactivity. The band of interest (indicated by the arrow) was selected based on its expected molecular weight (52~61 kDa).

Supplementary Table 1 Detailed results of GO enrichment analysis.
