# Supplementary figures and images for: Inhibition of CYP450 family 1 subfamily B member 1 (CYP1B1) expression in macrophage reduces the inflammatory response in type 2 diabetes mellitus combined with tuberculosis
Source: Front Endocrinol (Lausanne). 2025 Aug 21;16:1617292. doi: 10.3389/fendo.2025.1617292 (PMC12408331; doi:10.3389/fendo.2025.1617292)

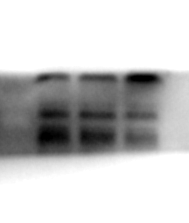

Supplement: Supplementary Figure 1 — Flow chart of this research. [file Image1.tif]

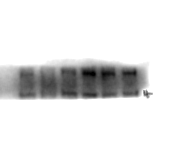

Supplement: Supplementary Figure 2 — Machine learning algorithms identifying key genes related to the mechanisms by which T2DM affects TB. (A) LASSO coefficient distribution plot. (B) LASSO coefficient profile plot, where the vertical dashed lines indicate the log(λ) values corresponding to the minimum mean squared error and one standard error away from the minimum mean squared error. The plot also shows the number of variables with non-zero coefficients in LASSO regression. (C) RF model achieving the highest accuracy at N=17. (D, E) SVM-RFE feature: 5-fold cross-validation accuracy plot and error plot. (F) Venn diagram showing the intersection of genes selected by LASSO, RF, and SVM-RFE. (G) Gaussian mixture model determining the logistic regression model pattern related to AUC values. [file Image2.tif]

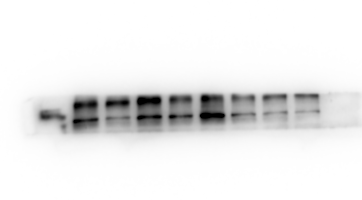

Supplement: Supplementary Figure 3 — (A) qPCR analysis of CYP1B1 mRNA expression following transfection with CYP1B1 SiRNA. (B, C) Western blot analysis showing the reduction of CYP1B1 protein expression after SiRNA-mediated knockdown. Note: Statistical significance is denoted as follows: ns, not significant (P > 0.05), *P < 0.05, **P < 0.01, and ***P < 0.001. The polyclonal anti-CYP1B1 antibody may produce non-specific bands due to its broad reactivity. The band of interest (indicated by the arrow) was selected based on its expected molecular weight (52~61 kDa). [file Image3.tif]

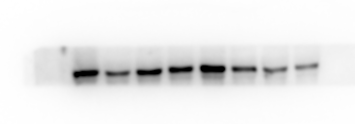

Supplement: Supplementary file 5 [file Image4.tif]

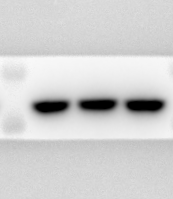

Supplement: Supplementary file 6 [file Image5.tif]

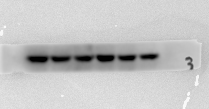

Supplement: Supplementary file 7 [file Image6.tif]

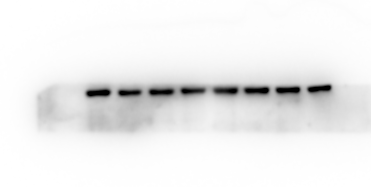

Supplement: Supplementary file 8 [file Image7.tif]

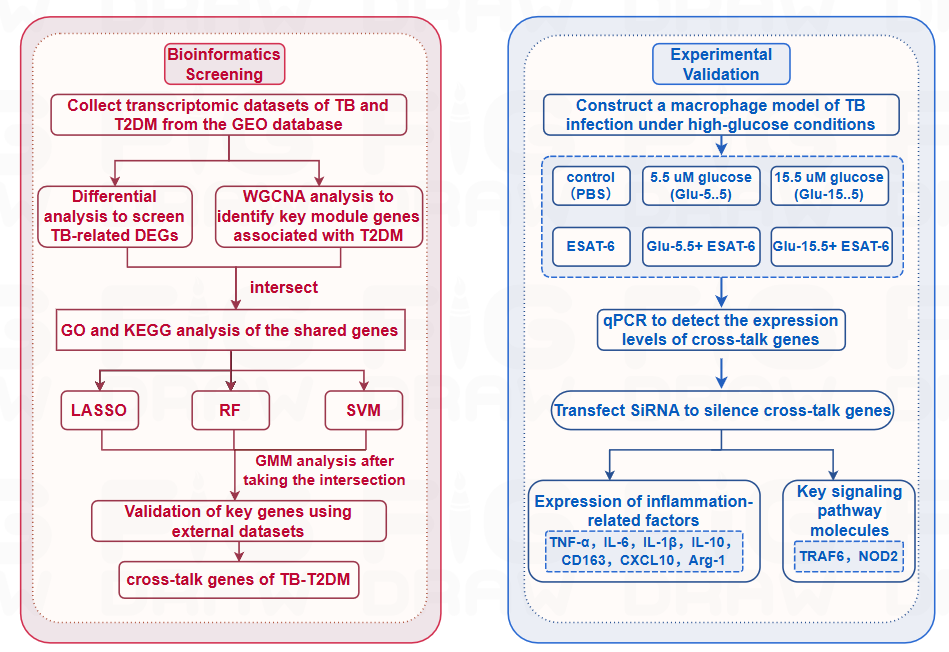

Supplement: Supplementary file 9 [file Image8.png]

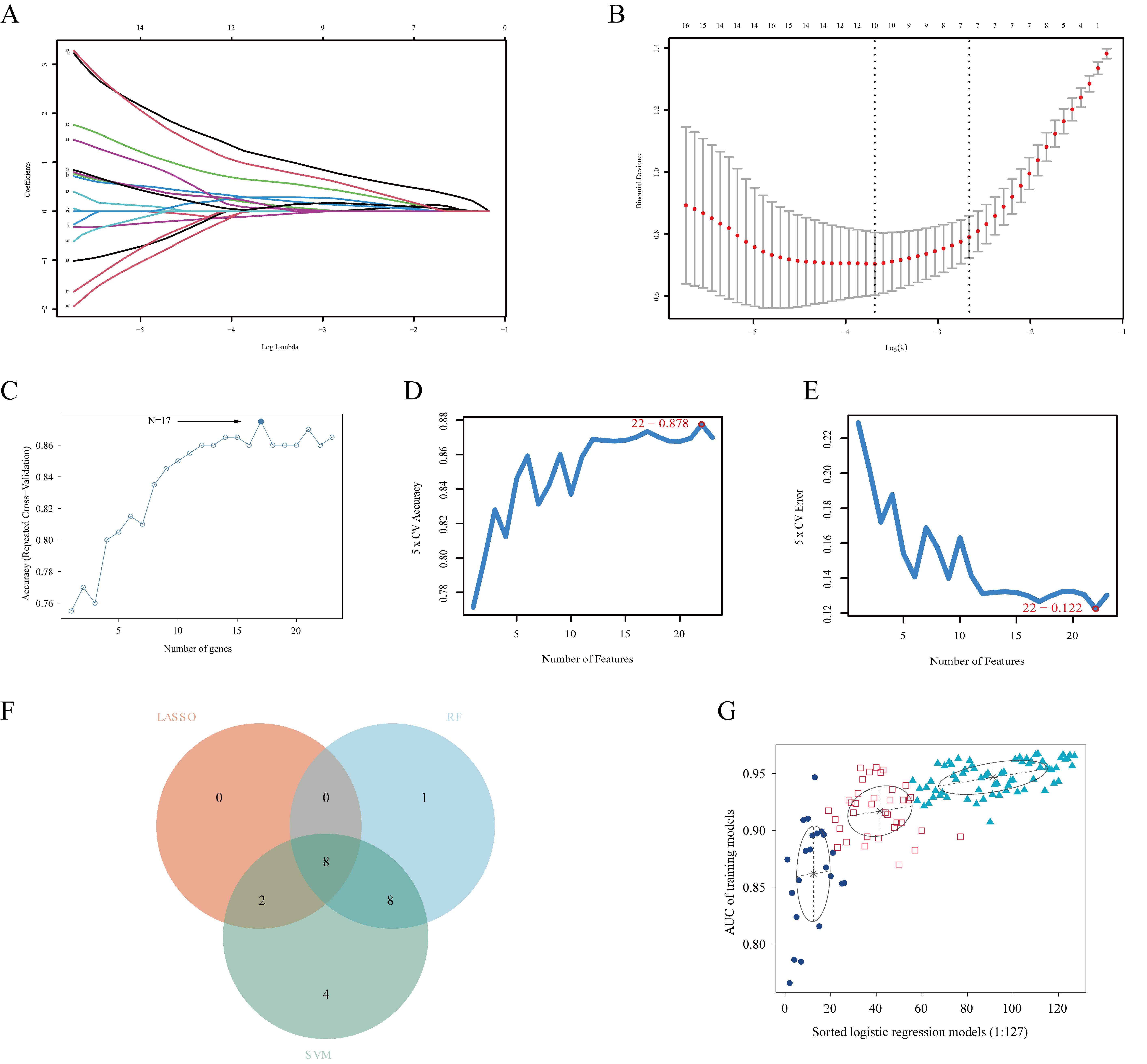

Supplement: Supplementary file 10 [file Image9.tif]

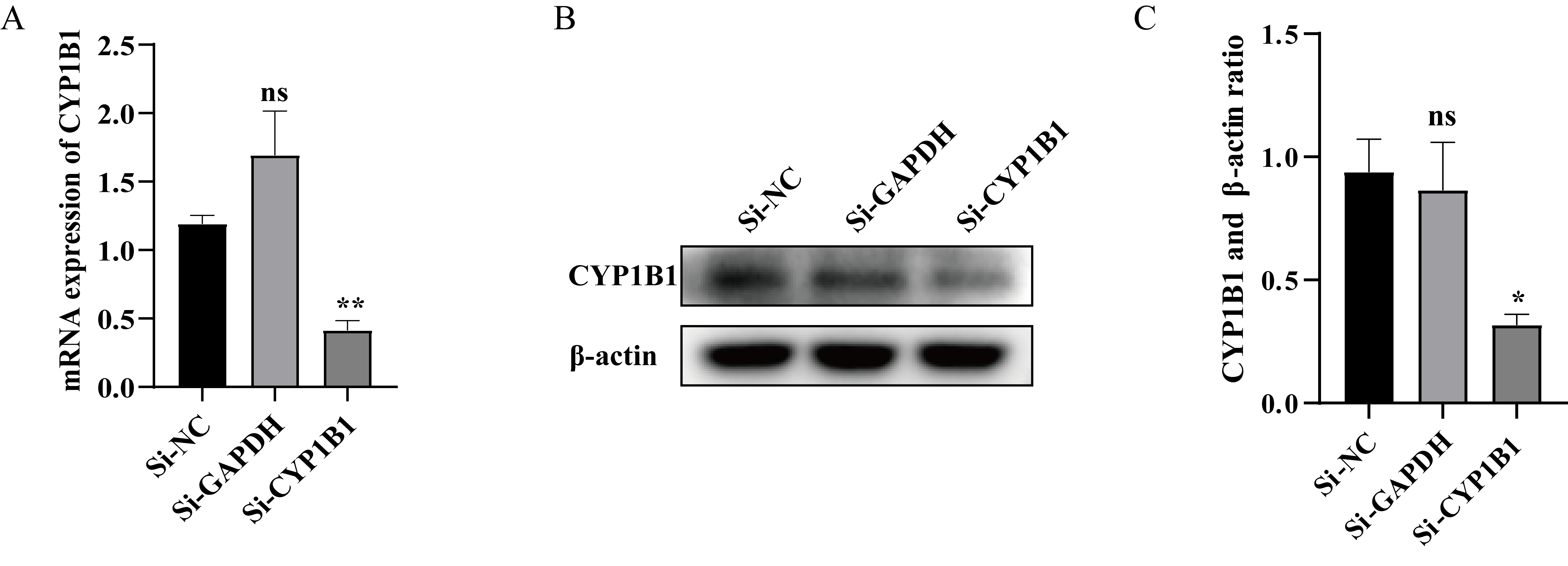

Supplement: Supplementary file 11 [file Image10.tif]
